# Supplementary figures and images for: Genomic diversity landscapes in outcrossing and selfing Caenorhabditis nematodes
Source: PLoS Genet. 2023 Aug 16;19(8):e1010879. doi: 10.1371/journal.pgen.1010879 (PMC10461856; doi:10.1371/journal.pgen.1010879)

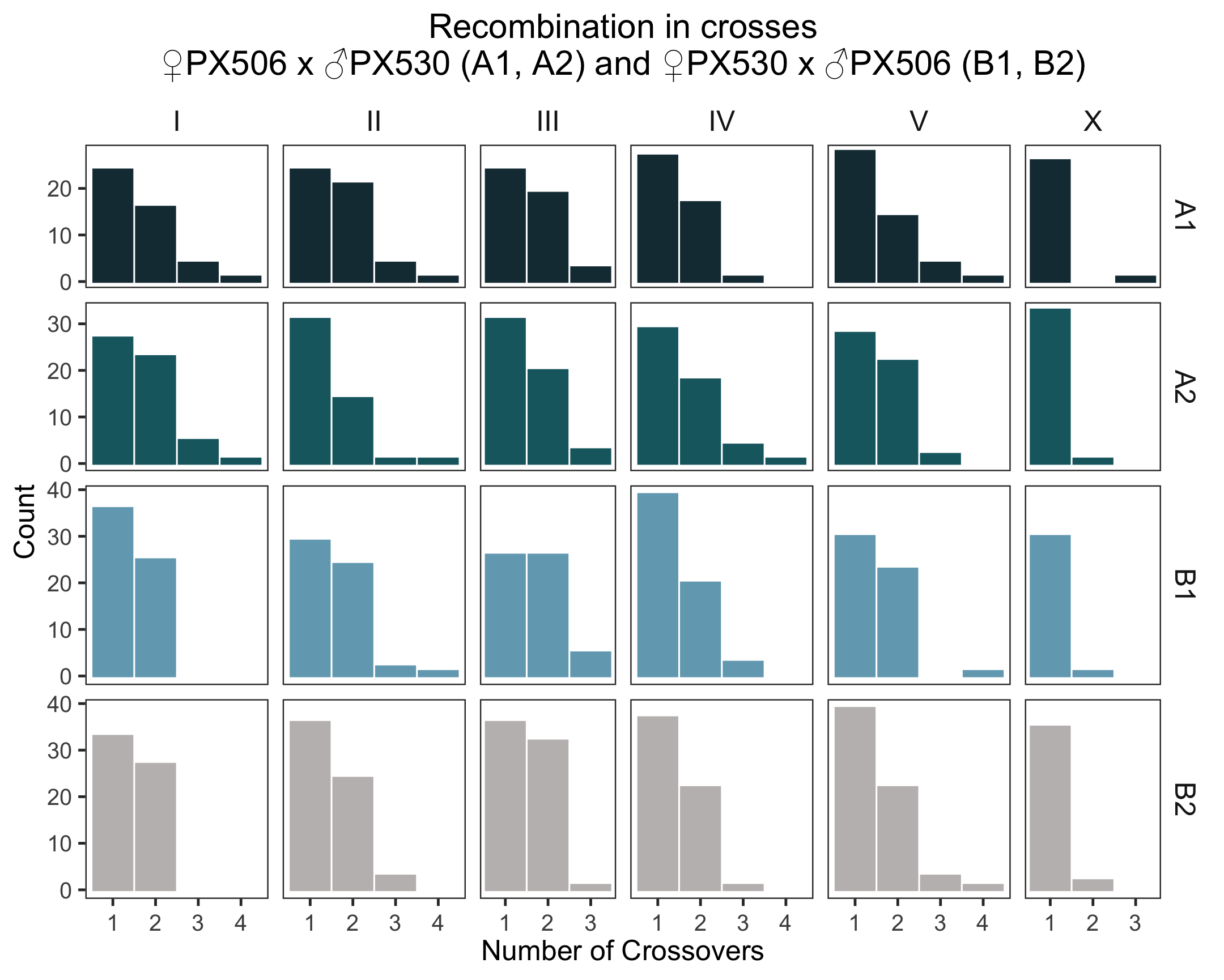

Supplement: S1 Fig — A1 and A2 are crosses of ♀ PX506 x ♂ PX553; whereas B1 and B2 are crosses of ♀ PX553 x ♂ PX506. We observed similar distributions of the number of crossover events in all crosses. Autosomes had more recombination events than the sex chromosome (X). (TIF) [file pgen.1010879.s001.tif]

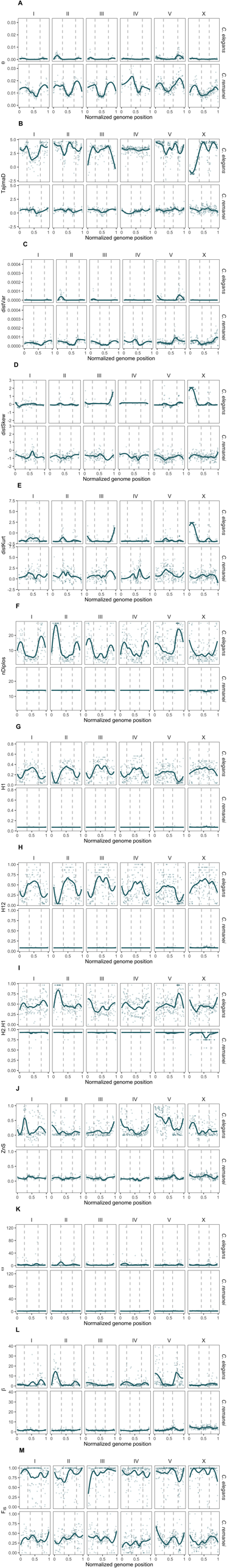

Supplement: S2 Fig — Dots represent the diversity statistics estimated in 100 kb non-overlapping windows, whereas lines show locally weighted smoothing of these values. Windows with less than 10% covered positions were removed from the analysis. The vertical dashed lines indicate the boundaries of regions of low recombination central domain. The x-axis represents the normalized genome position. See the description of statistics in the Methods section. In almost all statistics, C. remanei and C. elegans exhibit distinct patterns and scales. (TIF) [file pgen.1010879.s002.tif]

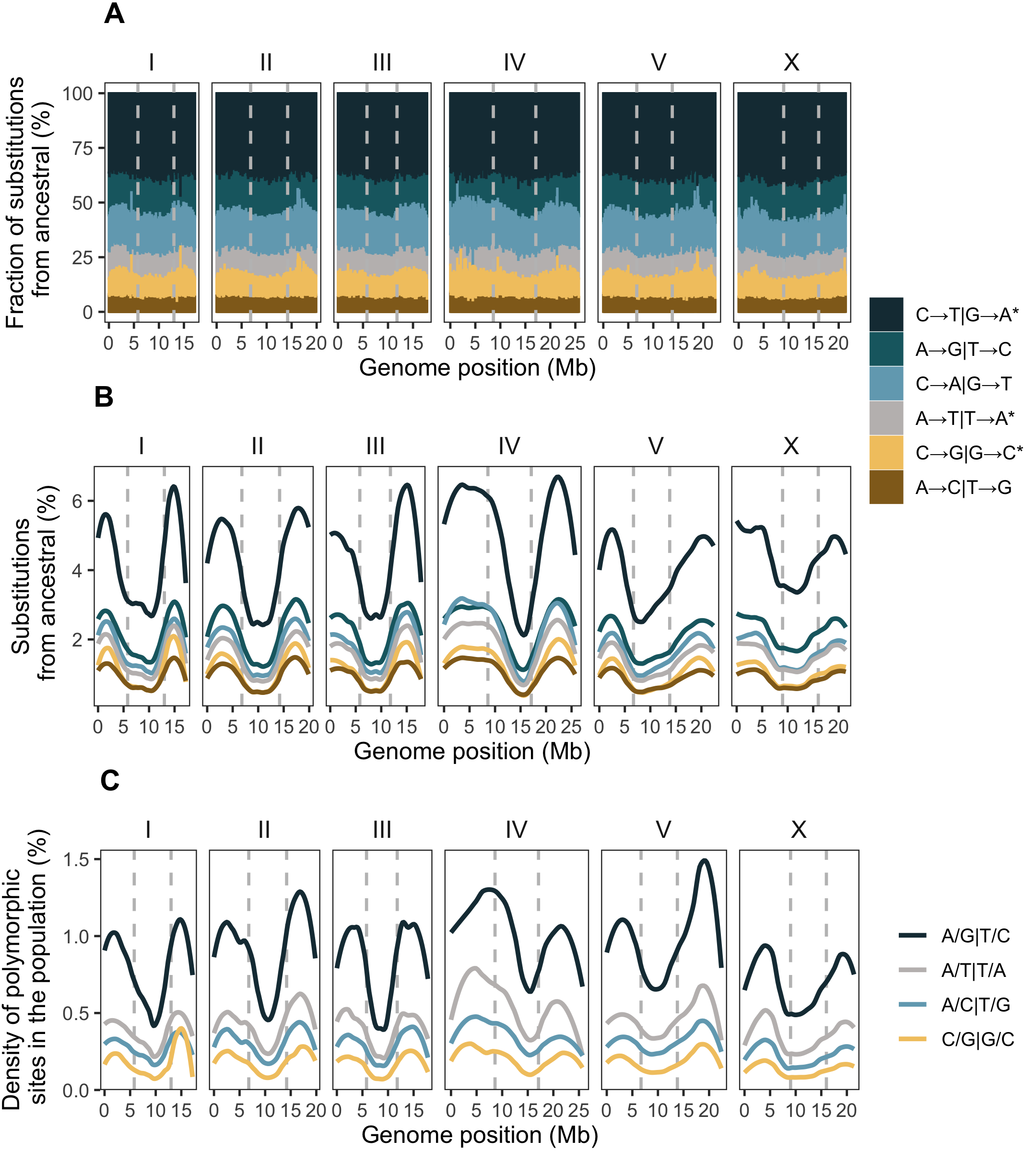

Supplement: S3 Fig — The first two lines (dark teal and teal) are transitions and the other lines are various forms of transversions. (A) Percent of substitutions for each class as estimated from ancestral GC content as a fraction of coverage of a 1 Mb genomic window. (B) Relative fraction of each substitution type at a given genomic location. Overall, relative proportions for three of the substitution types are homogeneous along the genome, while the three other types (C→T|G→A, A→T|T→A, and C→G|G→C, marked with asterisks) show small but significant differences between domains of recombination (see S2 Table). (TIF) [file pgen.1010879.s003.tif]

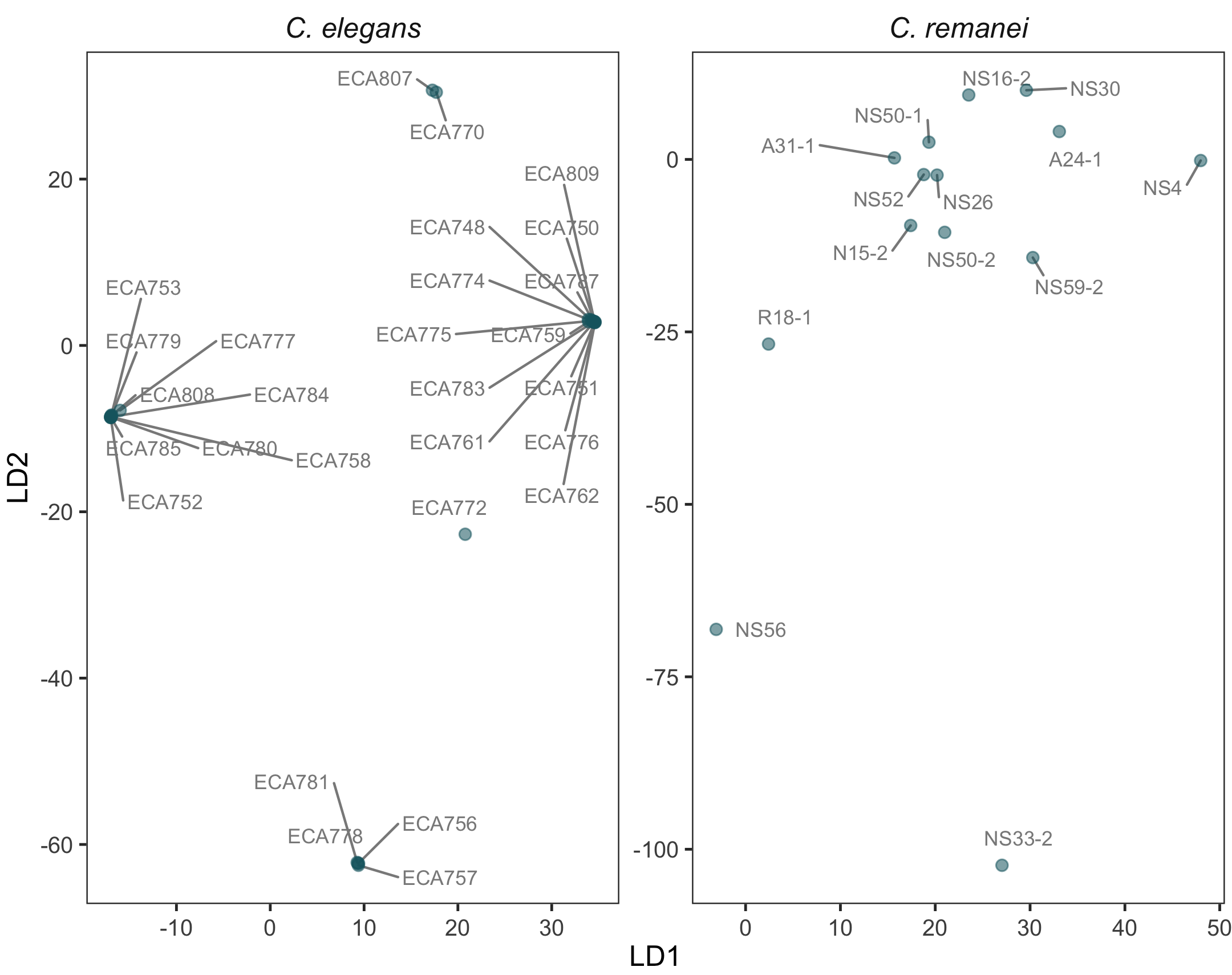

Supplement: S4 Fig — LD1 and LD2 show two latent dimensions. Some individuals in C. remanei population are closely related. In the C. elegans population, there are few lines with several individuals that are almost genetically identical and were combined to isotypes in previous studies ([81], see S3 Table). (TIF) [file pgen.1010879.s004.tif]

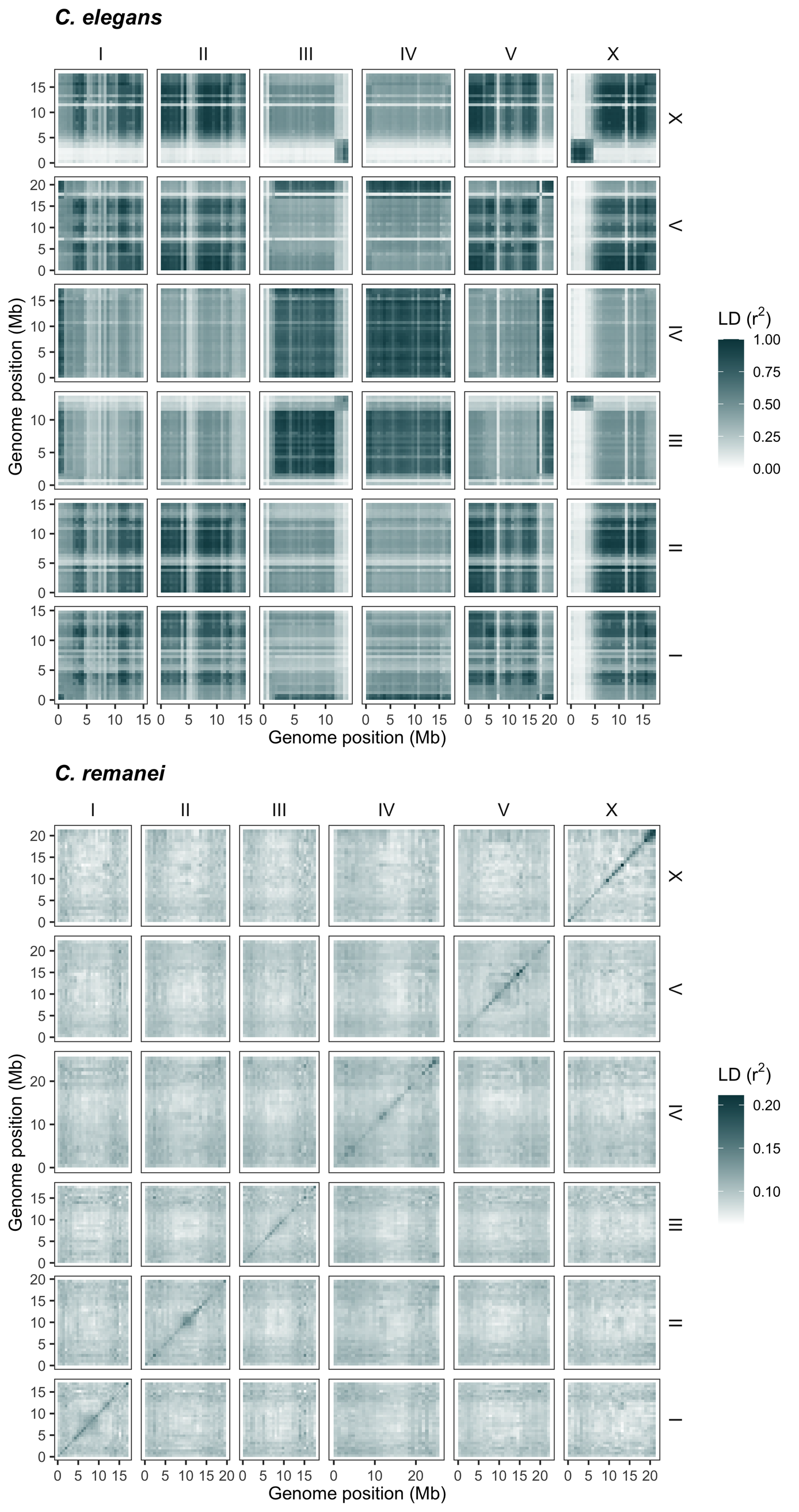

Supplement: S5 Fig — The panels show linkage disequilibrium (r2) in the C. elegans (A) and C. remanei (B) populations. The linkage between and within chromosomes is highly similar in C. elegans, but significantly different (see the main text). C. remanei shows the fast decay of linkage disequilibrium (Fig 4) and low interchromosomal LD. (TIF) [file pgen.1010879.s005.tif]

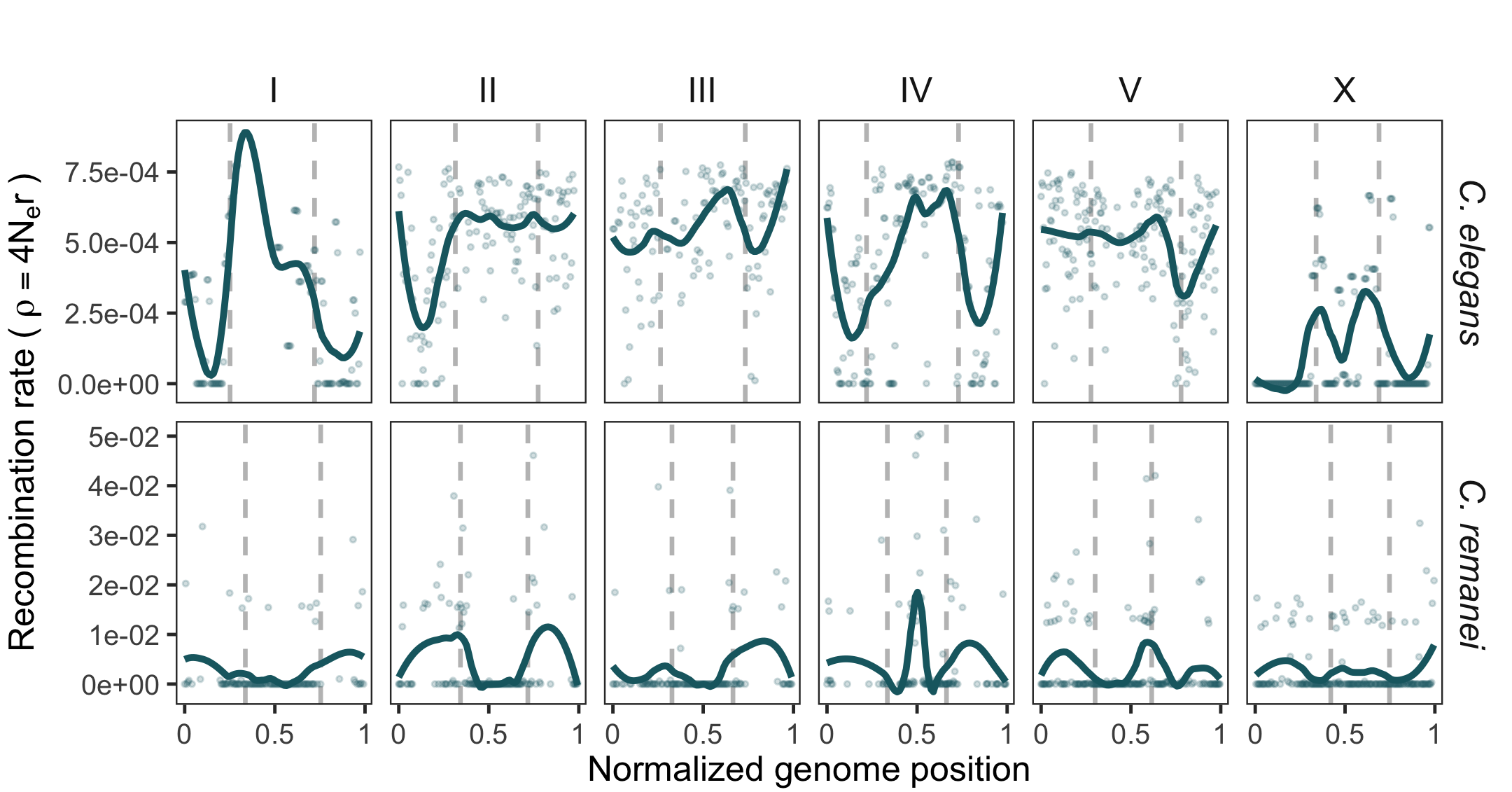

Supplement: S6 Fig — The x-axis shows the normalized genome position. The vertical dashed lines indicate the boundaries of central regions of low recombination obtained from genetic maps. (TIF) [file pgen.1010879.s006.tif]

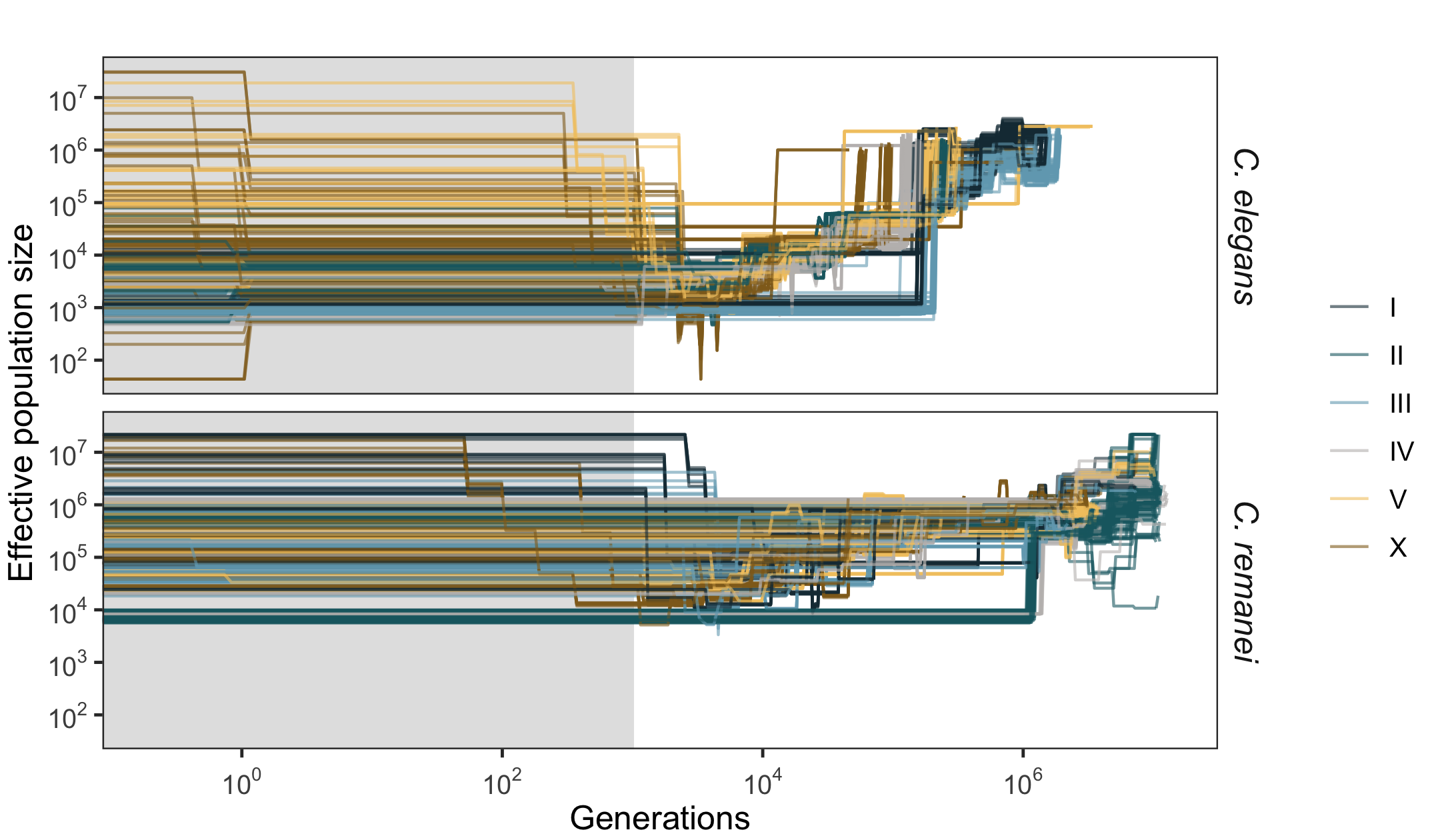

Supplement: S7 Fig — The color represents chromosomes. We ran 100 bootstrapped replicates using eight individuals from each species, each line represents one replicate. The grey shadow indicates the region of recent demographic history, where estimations are less accurate. We used one generation per year in this analysis and scaled of the mutation rate (x0.5) and coalescent time (x2) for C. elegans. (TIF) [file pgen.1010879.s007.tif]

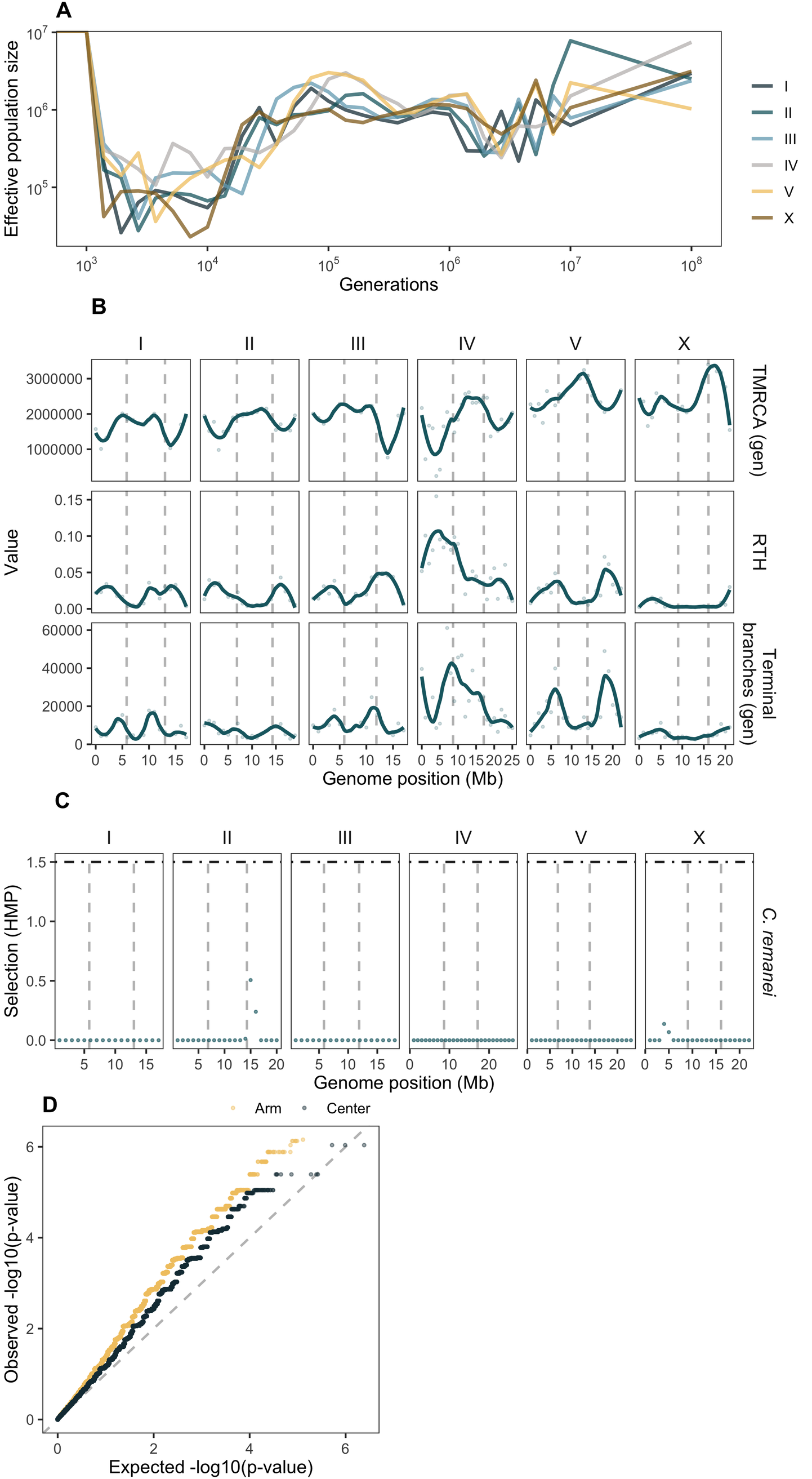

Supplement: S8 Fig — (A) Demographic history of the C. remanei population estimated for each chromosome. (B) Tree statistics calculated from the genealogies averaged for 100 kb windows; TMRCA (time to the most recent common ancestor), RTH (relative TMRCA half-time), and the lengths of terminal branches of the trees. (C) Signatures of positive selection along the genome, the y-axis shows the p-values after the correction on multiple comparisons using the harmonic mean approach (see the Methods). (D) Quantile-quantile plot displays p-values from the tests for positive selection (y-axis) versus the expected uniform distribution of p-values (x-axis). The yellow color shows sites on the arms, and the black color indicates sites on the central parts of chromosomes. (TIF) [file pgen.1010879.s008.tif]

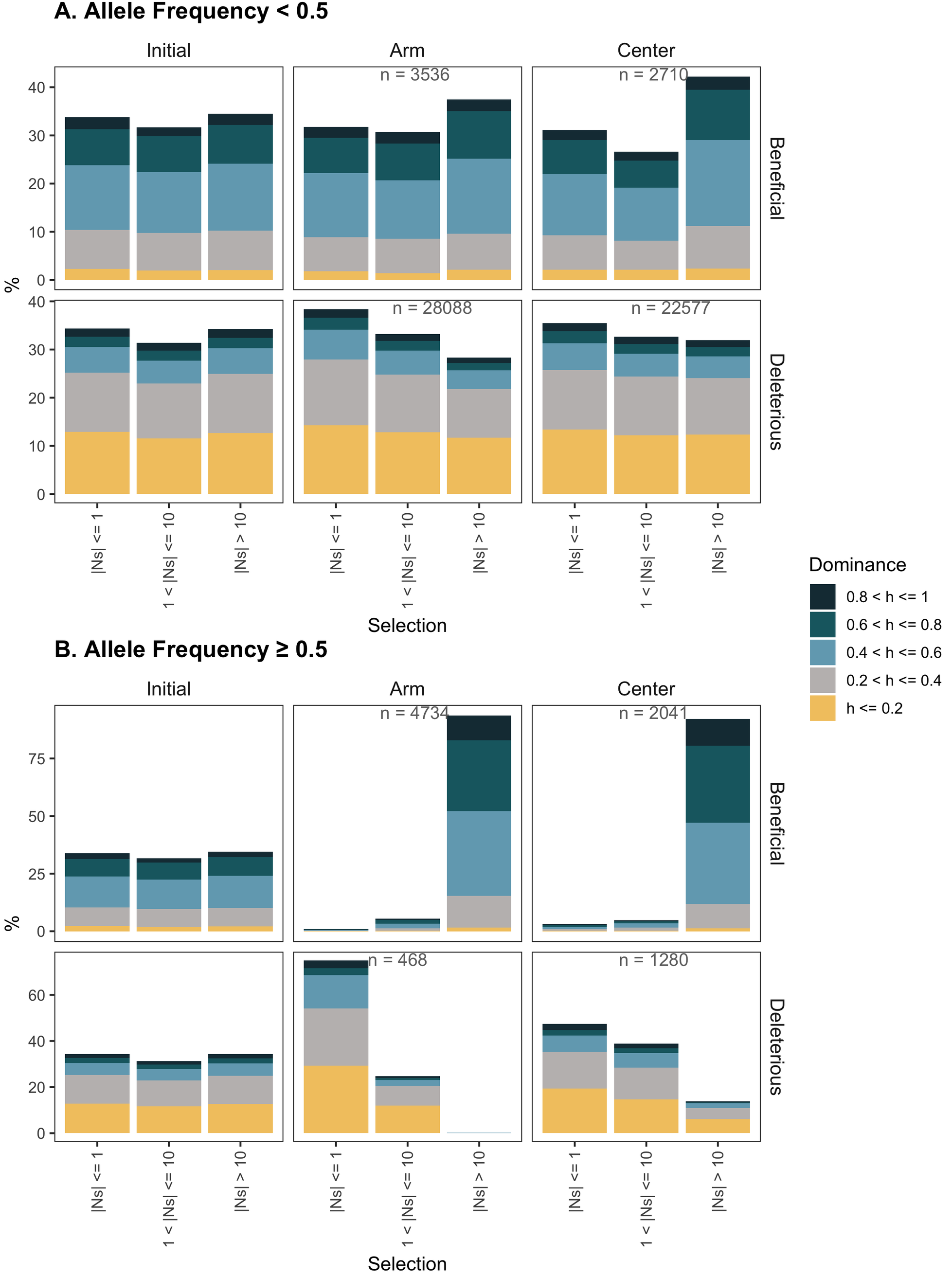

Supplement: S9 Fig — This picture depicts mutations from the SD&SB-SD&SB class described in S4 Table with the uniform mutation landscape. (A) Percentage of mutation classes with allele frequency more than 0.5 at the beginning of the simulation (“Initial”) and the end on the arms and centers. Colors display the class of dominance coefficient (h), and the columns represent the strengths of selection (absolute values of Ns, where N is the population size of 5,000 and s is the selection coefficient). (B) The percent of corresponding mutation classes of mutations with allele frequency less than 0.5. (TIF) [file pgen.1010879.s009.tif]

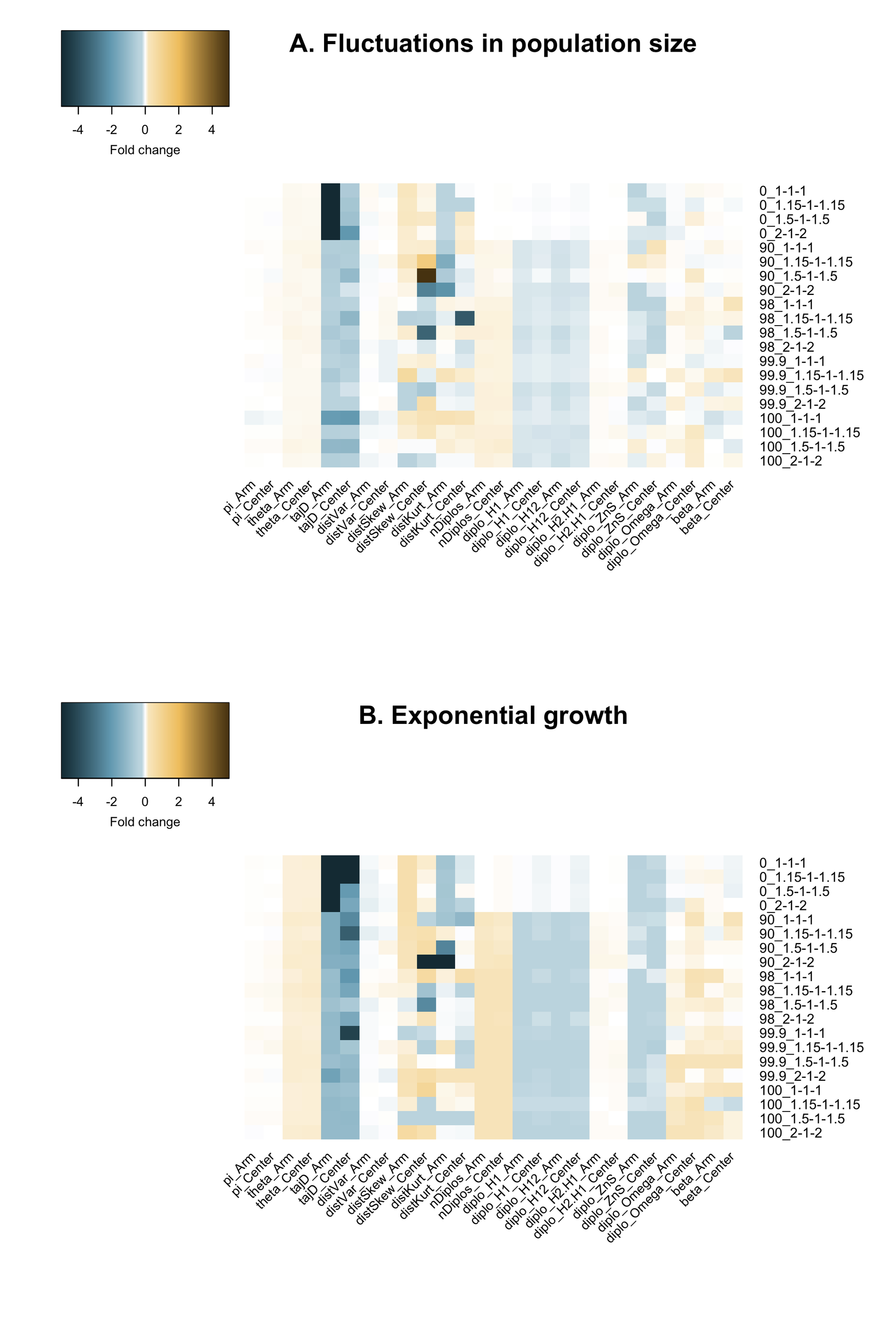

Supplement: S13 Fig — The colors represent the fold change in statistics at the end of the simulation versus before changes in size. (A) Fluctuation in population size for 100 generations, where every five generations, the population size went from 5,000 to 15,000 and then back. (B) Exponential growth of 3% for 100 generations. (TIF) [file pgen.1010879.s013.tif]

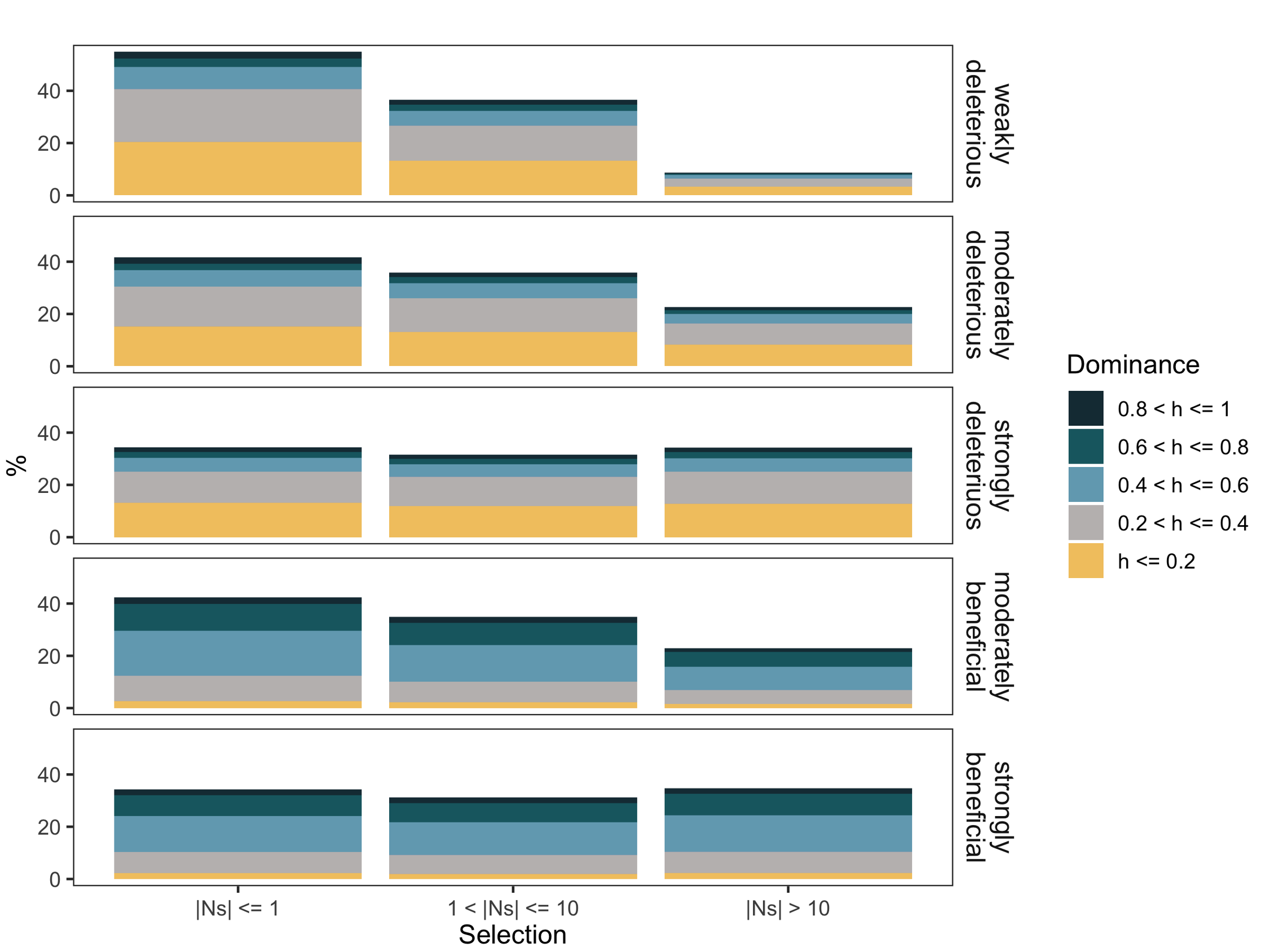

Supplement: S14 Fig — The columns show the percentage of each class of selection coefficients drawn from gamma distributions with different parameters (see S4 Table). Dominance coefficients were chosen independently from a mixture of uniform and beta distributions with distinct parameters for deleterious mutations and beneficial mutations (see the Methods and SLiM scripts at https://github.com/phillips-lab/CR_CE_popgen/tree/main/simulations). (TIF) [file pgen.1010879.s014.tif]
