# Supplementary material for: Genomic diversity landscapes in outcrossing and selfing Caenorhabditis nematodes
Source: PLoS Genet. 2023 Aug 16;19(8):e1010879. doi: 10.1371/journal.pgen.1010879 (PMC10461856; doi:10.1371/journal.pgen.1010879)
Supplement: S1 Data Appendix A1 — We describe an approach to classify evolutionary simulations and empirical data by evolutionary scenarios using deep learning, its application, caveats and future directions. (PDF) [file pgen.1010879.s019.pdf]

Appendix A1: Additional analysis, classification of simulated and empirical data using convolutional neural networks

An approach for classification of simulated and empirical data by evolutionary scenarios

Deep learning is an important emerging approach to use on massive and heterogeneous data to capture and classify hidden dependencies and patterns, a machine learning technique that is already finding applications in functional genomics ([1], [2], [3]), medicine ([4], [5]), and population genetics ([6], [7]). In this work, we conducted numerous simulations to study the pattern of diversity statistics under various evolutionary scenarios on genomic landscapes with nonuniform recombination rate, mutilation rate, different rate of selfing in population, inbreeding, combinations of neutral, deleterious and beneficial mutations. Given the large number of parameters and observations involved, we opted for a deep learning approach to discern the effects of those evolutionary forces and employed convolutional neural networks to classify evolutionary scenarios in simulated and empirical data.

In order to classify mutation rate, selfing rate, and type of selection on empirical data we trained a convolutional neural network (CNN) on a variety of features from the simulated chromosomes. Each simulated chromosome was divided into 75 sub-windows and 13 population diversity statistics were calculated within each sub-window, resulting in 13 by 75-pixel images used as input features for the CNN. The targets for the network consisted of one-hot encodings on each combination of the three evolutionary scenarios.

PCA and saliency plots were employed to visualize the similarity between simulated and empirical features. We projected the empirical features to PC space fitted to the simulated features in order to see how close the simulations resemble the empirical features. Saliency plots allow us to glimpse the features through the lens of the CNN. That is, we can see how sensitive the network classification is to perturbations of the summary statistics in each sub-window.

The CNN architecture was composed of four convolutional layers and four dense, fully connected layers. To prevent overfitting, dropout was used in between all but the final two layers. ReLU activations were used on all but the final layer, which had linear activation to improve numerical stability for the categorical cross-entropy loss functional. Weight learning was controlled by the Adam optimization routine with an initial learning rate of 1.5e-5. To reduce overfitting, we stopped training after the validation loss improved no more than 1e-7 for 100 epochs and saved the weights that resulted in the highest validation accuracy. The CNN was implemented using the Keras module within TensorFlow ([8]). The details of the analysis are at https://github.com/phillips-lab/CR_CE_popgen/cnn_model/.

Classification of simulated data

Because multiple genetic and evolutionary processes can lead to similar outcomes in terms of genomic diversity (e.g., mutation vs. recombination), we sought a comprehensive approach toward distinguishing major factors structuring genetic variation. We, therefore, trained a convolutional neural network on nine diversity statistics (π, θ, Tajima's D, variance, skew, kurtosis, ω, and β -statistics) with the intent to classify population characteristics such as selfing rate, mutation landscape, and selection, and then apply this network to our empirical samples for *C. elegans* and *C. remanei*. The network was trained on the first set of simulations described above, with statistics estimated on 40-kb genomic sliding windows and normalized for each simulation. Unfortunately, haplotype-based statistics had to be excluded because they could not be scaled using fixed window size approaches given the drastically different estimated effective population sizes of *C. elegans* and *C. remanei*. Additionally, we excluded simulations with balancing selection as they were mostly classified as outcomes including deleterious and beneficial mutations.

Overall, the network did well classifying simulation results under most parameter combinations (A1.1 Fig). Matthews correlation coefficients that show the accuracy of predictions of unbalanced classes for joint-feature predictions was 51.8%, with specific predictions for mutation landscape being 67.7%, selfing rate being 85.2%, and selection regime being 71.7%. The confusion matrix is in A1.2 Fig. The largest point of error for the network was generated by subtle differences in mutation rate across chromosomal regions, with the network confusing the uniform mutation rate with the 15% increase in mutation rate on the arms (A1.2 Fig). Many of the population genetic statistics are quite similar for these scenarios (S10 Fig). Other cases that confused the network are distinguishing between simulations with neutral mutations and a combination of neutral and deleterious mutations. This is undoubtedly because even in the deleterious mutation case, many of the mutations are drawn from a distribution in which about a third of the selection coefficients have Ns <1 (S9 Fig). Also, many deleterious mutations tend to have

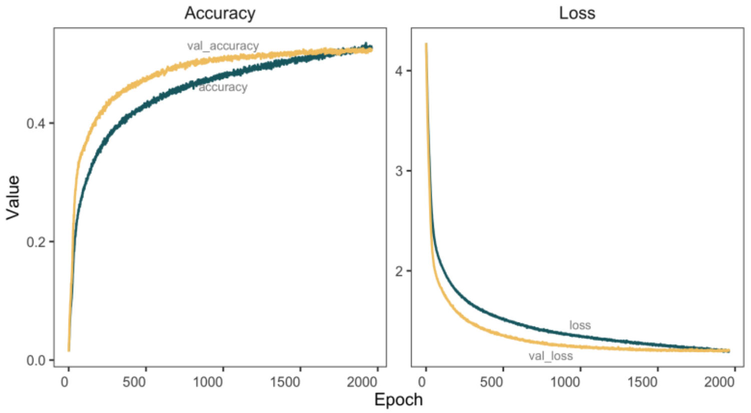

A1.1. Fig. Model accuracy and cross-entropy loss curves for the convolutional neural network.
The orange color shows the validation accuracy or loss, and the teal color indicates values for training.

short persistence times, so those present in a given population tend to be recessive and effectively neutral when rare.

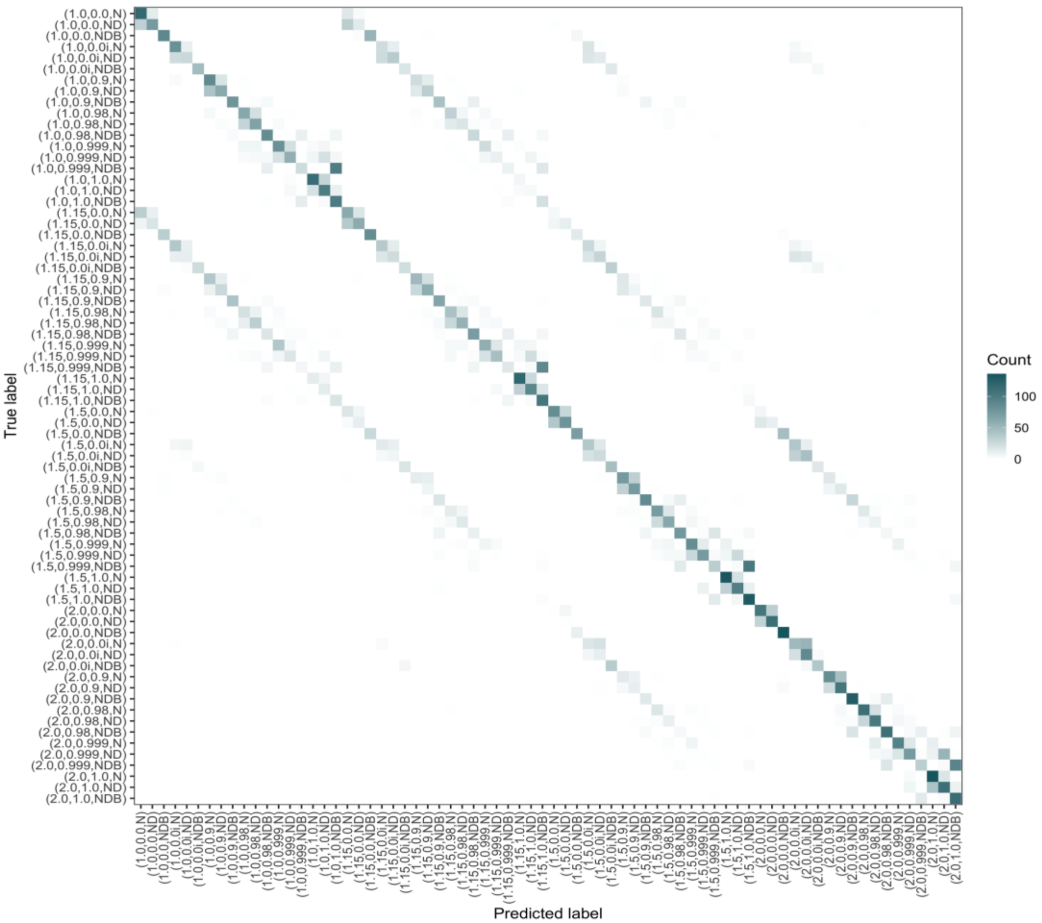

A1.2 Fig. **The confusion matrix for the convolutional neural network.**

The color represents the count of classified simulations. The values on the axes represent the simulated evolutionary scenario, where the first number denotes the mutation rate increase on the arms relative to the central domains, e.g., 1.5 is 50% more mutations on the arms. The second values show the selfing rate, where "0.0" stands for the outcrossing populations, "0.0i" are outcrossing with inbreeding at the end of simulations, and for instance, 0.9 is 90% selfing in the population. The third value indicated the selection regime, with "N" for neutral simulation, "ND" for neutral and deleterious, and "NDB" for neutral, deleterious, and beneficial mutations. Most values are located around the main diagonal, where the predictions match the simulated scenarios. The two off-diagonals indicate confusion between the adjacent mutation rates. For instance, when the mutation rate on the arms was 15% higher the network sometimes predicted either the uniform or 50% more mutations on the arms. Additionally, there is a confusion around some of the neutral and neutral/deleterious scenarios (as seen by 2x2 blocks on the diagonals), probably because the fraction of nearly-neutral mutations in some simulations is high (see S9 Fig and A1.1 Fig).

Classification of empirical data

Then we classified the normalized diversity patterns from the empirical data from *C. elegans* and *C. remanei* populations using the network trained on the simulations (A1.3 Fig). For *C. elegans*, predictions tended to differ per chromosome. In each case, the selfing rate was predicted to be less than 100%, and some amount of effective recombination was detected, which is consistent with our estimation of outcrossing rate directly from linkage disequilibrium. For whatever reason, Chromosome I from this population of *C. elegans* has a qualitatively different pattern of variation that is more consistent with frequent historical outcrossing and more recent self-reproduction. One of the many possibilities is that it may be connected to the *zeel-1*/*peel-1* system, located on Chromosome I, which is responsible for gametic incompatibility and known to be under balancing selection ([9]). For *C. remanei*, all chromosomes were unexpectedly classified as adhering to one of the selfing scenarios. This is surprising because the network performed very well in distinguishing among outcrossing rates in the simulations.

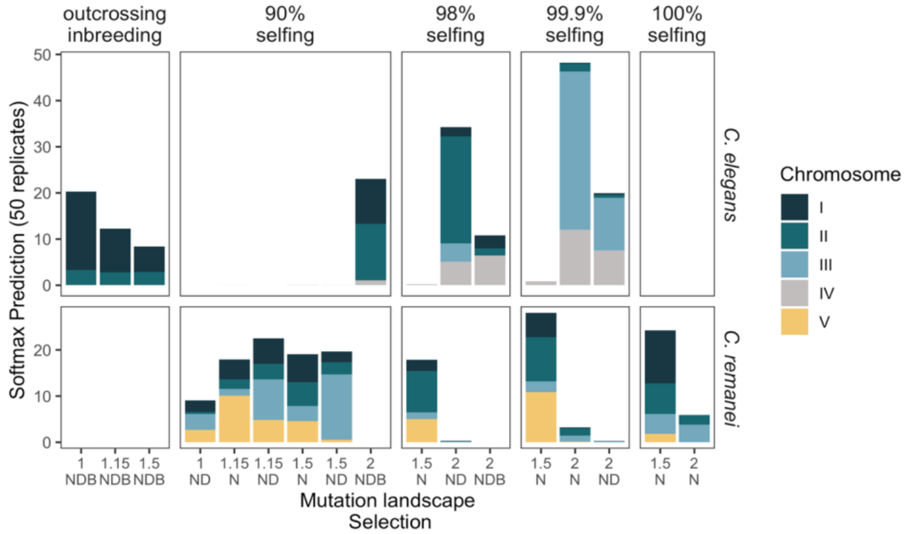

A1.3 Fig. Classification of the empirical landscapes of *C. elegans* and *C. remanei* by evolutionary scenarios.

The predictions show the probability (softmax) of each bootstrapped replicate of a chromosome to belong to a specific class of evolutionary scenarios. Only classes with more than 5 in total are shown. Colors represent the bootstrapped replicates of chromosomes; for each nematode, we used only 4 autosomes. Columns correspond to the selfing rate, where “outcrossing inbreeding is outcrossing populations that underwent a bottleneck, and the percentage of selfing defines the selfing rate in simulated populations. The classes represent mutation landscape (“1” – uniform; “1.15” – 15% more mutations on the arms; “1.5” – 50% more mutations on the arms; “2” – 2 times more mutations on the arms. “N”, “ND”, “NDB” mean neutral, neutral, and deleterious, and neutral, deleterious, and beneficial selection regimes. All of the predictions indicate some amount of outcrossing in *C. elegans*. *C. remanei* was not classified as an outcrossing species, which it undoubtedly is. Misclassification might be caused by the complex demographies of nematode populations and/or incompleteness of genomic data, especially on the arms.

To explore these differences a bit more fully, we analyzed the distribution of the simulation and empirical sample statistics using principal component analysis (A1.4 Fig). In keeping with the above, positions of the *C. elegans* chromosomes in the principal component space tended to be dispersed but within the range of simulated outcomes. In contrast, *C. remanei* tended to be located close together, indicating the similarity of population genetic processes but were at the edge of the scenarios captured by the simulations (A1.5 Fig). This would seem to indicate other demographic processes not included here have influenced the subtle signals being captured by the population genetic statistics. This conclusion is reinforced by the fact that saliency maps for the network (A1.6 Fig) show that even small perturbations in theta, kurtosis, and skew in the central domain are enough to change the predictions for outcrossing simulations. That means that the neural network is sensitive to small differences in the ratio of diversity among domains of recombination.

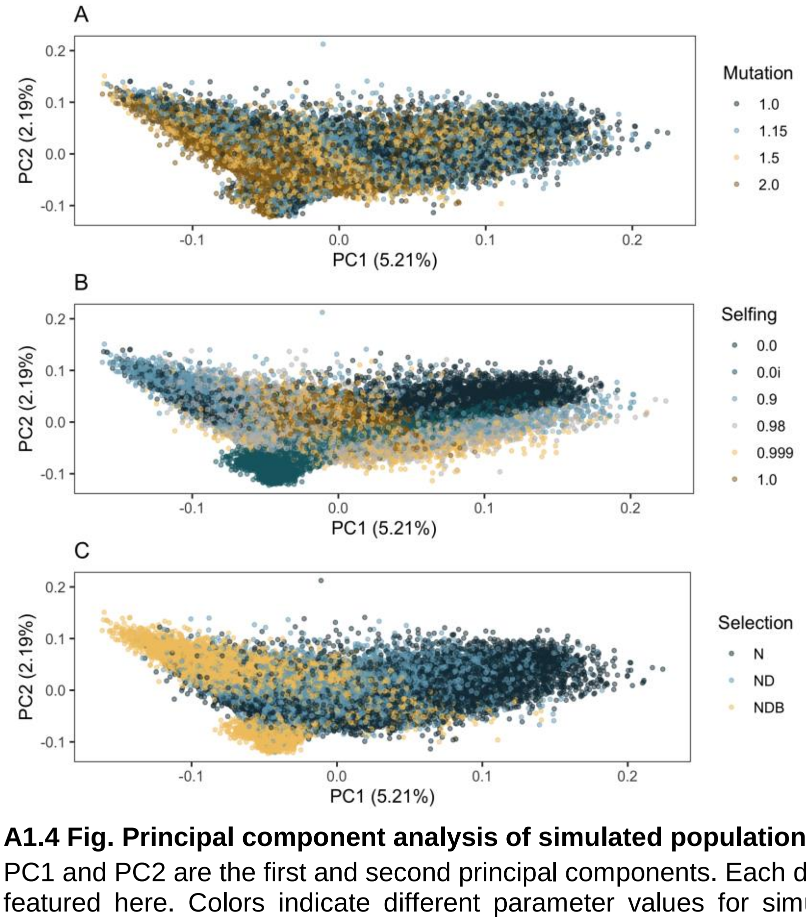

A1.4 Fig. Principal component analysis of simulated population.

PC1 and PC2 are the first and second principal components. Each dot shows one simulation from 41,840 featured here. Colors indicate different parameter values for simulation scenarios. **(A)** Mutation rate variation, where "1" is the uniform mutation rate, and "1.5" is 50% more mutations on the arms relative to the central domains. **(B)** Selfing rate in the simulated population, "0.0" denoted the outcrossing populations, "0.0i" outcrossing with inbreeding at the end of simulations, and for instance, "0.999" represents 99.9% of selfing in the population. **(C)** Selection regime. "N" shows neutral simulations, "ND" is neutral and deleterious mutations, and "NDB" is neutral, deleterious, and beneficial mutations.

The evolutionary simulations examining recent fluctuations of population size discussed above show that these fluctuations can in principle generate large changes in most of the diverse statistics used in the network classification. It is therefore likely that the main confounder for the *C. remanei* network analysis is demography (see S13 Fig and Fig 5). Unfortunately, there is an effectively infinite number of possible demographic scenarios involving population size fluctuations, and we did have enough computational resources for full exploration of these models at full population size, as such simulations are extremely memory and time-consuming. Refinement of the network model thus awaits additional empirical information and/or an expanded analytical framework.

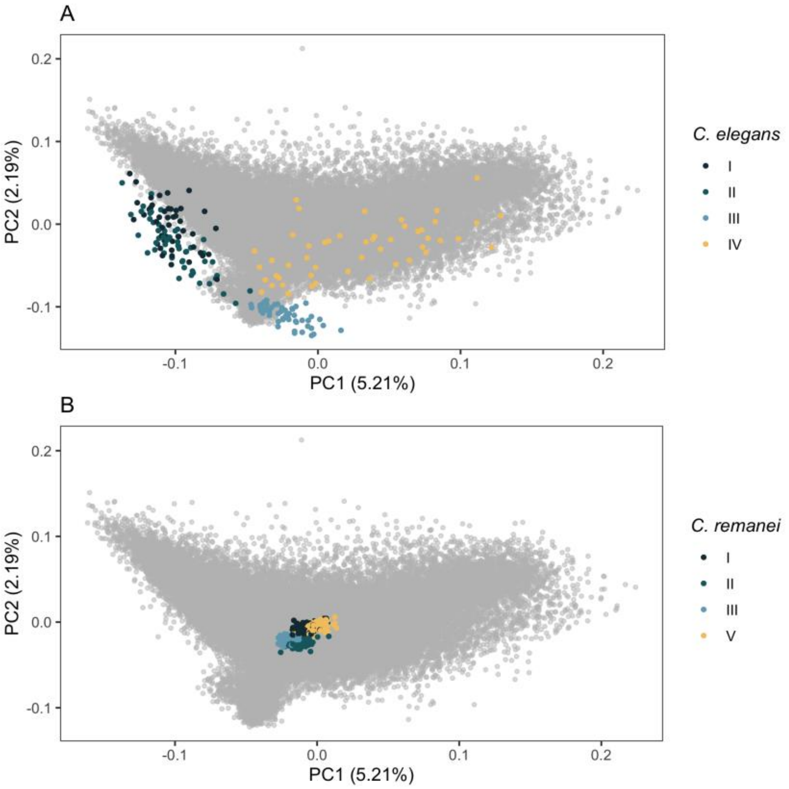

A1.5 Fig. Principal component analysis of simulated populations and position of chromosomes of *C. elegans* and *C. remanei* projected to the PCA space.

For each chromosome, we did 50 bootstrapped replicates sampling statistics from domains of low and high recombination. Colors indicate the chromosomes of *C. elegans* **(A)** and *C. remanei* **(B)**. Sex chromosomes and some autosomes with long tracts of repetitive sequences were removed from the analysis.

Finally, at the whole genome level, choices made during bioinformatic processing can also potentially subtly affect diversity statistics in a way that the network might not see. For instance, when filtering the sequence data, even small changes in filtering parameters, like changing the threshold for invariant sites can shift most of the diversity statistic estimations for *C. remanei* (A1.7 Fig).

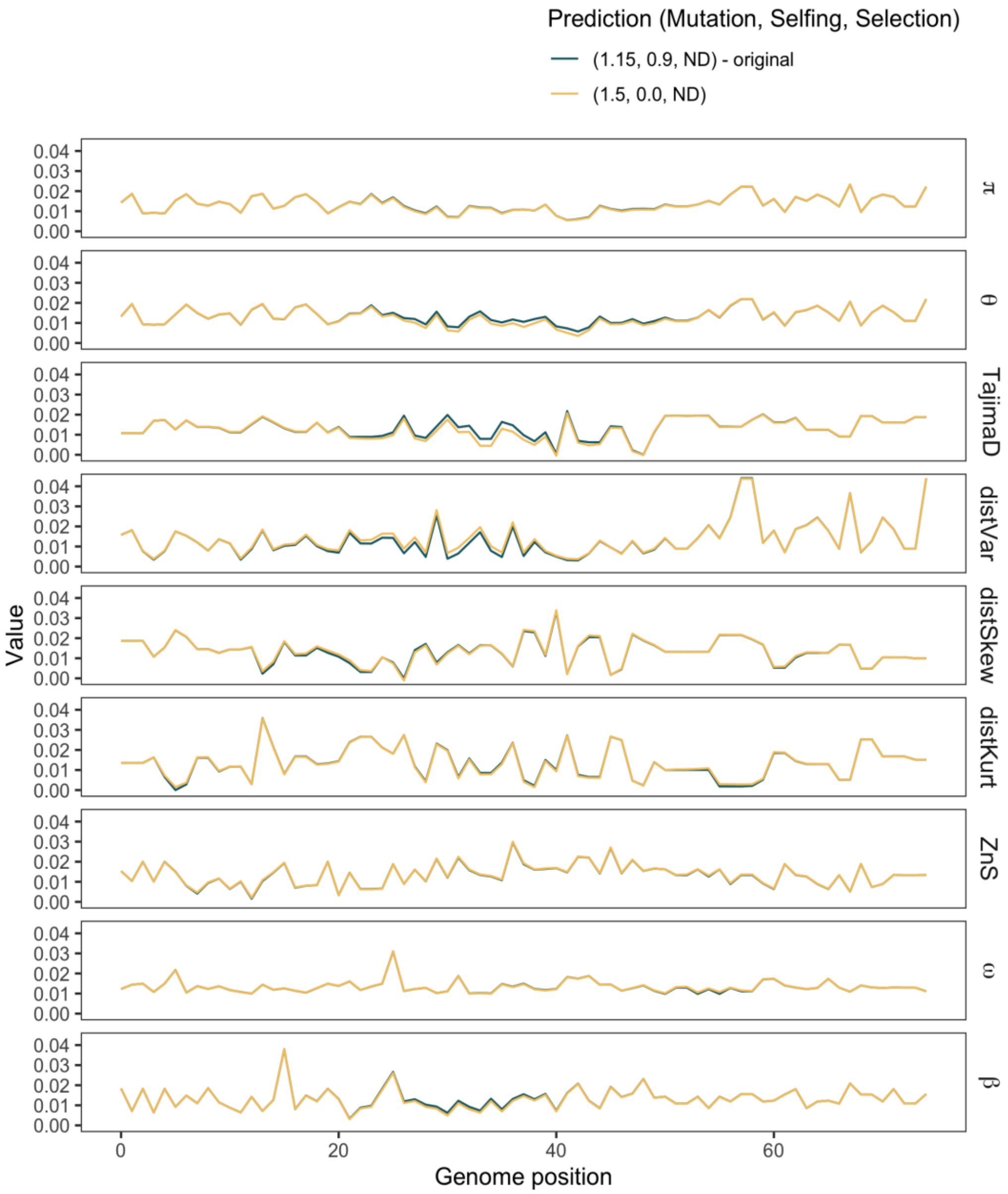

A1.6 Fig. Saliency plot for one of the bootstrapped replicates of chromosome I from the *C. remanei* population by the neural network.

The teal color shows the original diversity landscapes of 9 statistics. That replicate was classified as (1.15, 0.9, ND), meaning 15% more mutations on the arms, 90% selfing, and neutral and deleterious mutations. The orange color shows perturbed statistics, slightly changing to the original ones. However, that population would be classified as (1.5, 0.0, ND), with 50% more mutations on the arms, outcrossing with neutral and deleterious mutations. This example demonstrates how sensitive the model is to slight changes in diversity statistics.

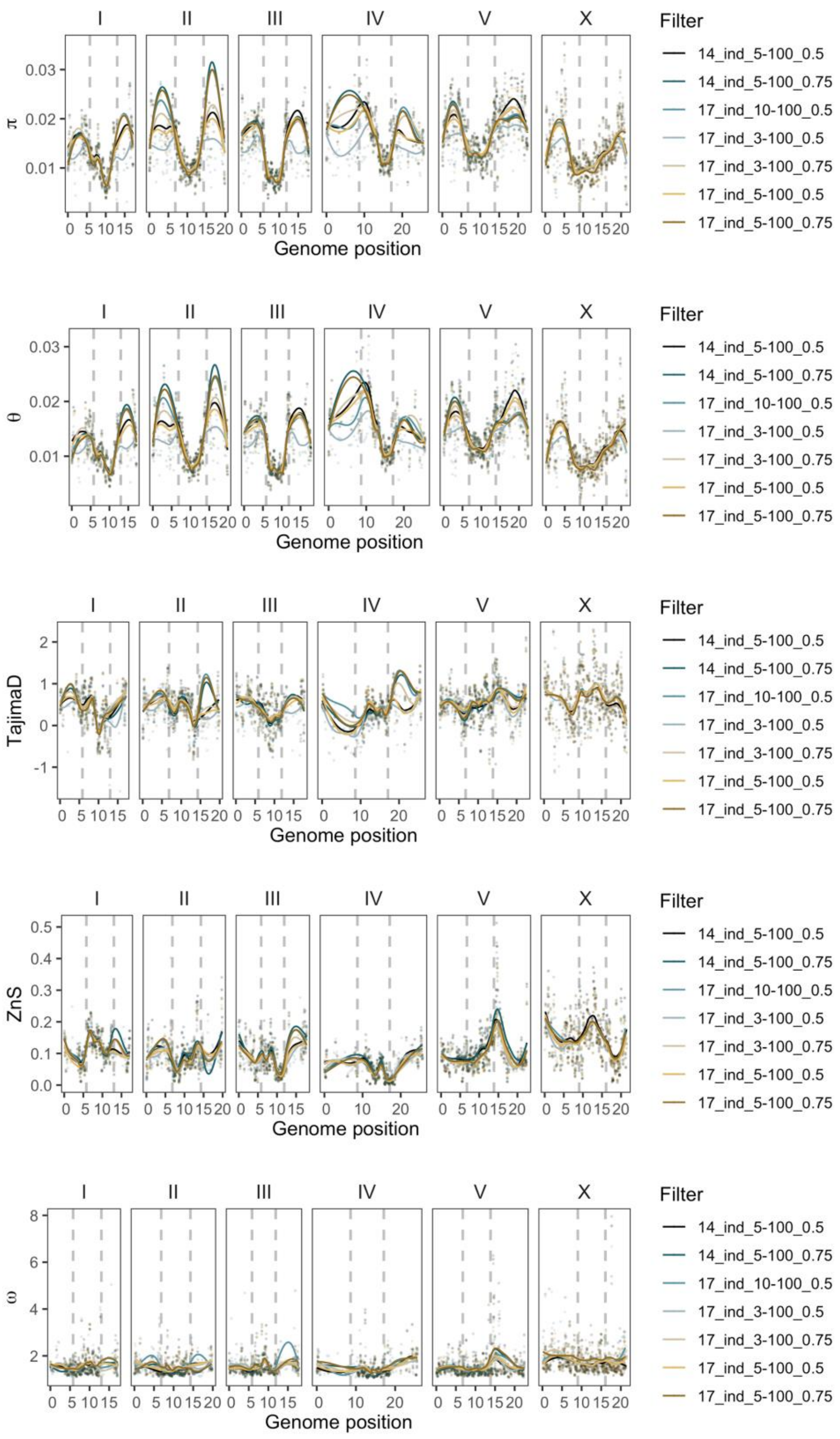

A1.7 Fig. Effects of bioinformatic filtering of the empirical data to the estimated diversity statistics. The colors indicate different parameters of the filters. The first value of the filter specifies the number of *C. remanei* individuals, either 14 or 17. The following two values are, correspondingly, minimal and maximal coverage, and the last is the fraction of individuals with the specified coverage. In the analysis, we used the top filter indicated by black color.

Caveats and future directions

We used neural networks to classify various potential evolutionary scenarios using known patterns generated by our forward-time evolutionary simulations that varied patterns of selection, selfing rate, and mutation landscape. Here, perhaps not surprisingly, selfing rate led to the most effective means of classifying potential outcomes. Overall, however, we were not particularly successful in classifying the specific empirical patterns observed in these species (Fig A1.3). Specifically, whereas patterns for *C. elegans* worked well from the viewpoint of our simulation results, those from *C. remanei* did not, as its patterns of genomic variation were frequently classified as being consistent with selfing rather than outcrossing. Particularly important here seems to be the fact that even small changes in the ratios of some statistics between domains can alter the predicted structure (see an example in A1.6 Fig; also, Figures S10, S11, S12).

Several factors could make our classification scheme particularly sensitive to these issues. First and foremost, we could not simulate all possible demographic histories of the population, because simulating populations of equivalent size to natural populations, as well as including large-scale changes in population size, turns out to be computationally infeasible. Nevertheless, it is clear that these effects can be substantial, as additional simulations of both recent size fluctuation and moderate exponential population growth showed that recent changes in the population size affect most of the statistics used for classification (S13 Fig). Further, even subtle factors, such as the quality of genomic coverage and bioinformatic assumptions, can lead to significant differences among the diversity statistics (A1.7 Fig). Long-read data may help close the gap in the coverage and improve the mapping quality for species with such high diversity as we see with these outcrossing nematodes. Lastly, it is possible that the precision of the classification could be improved by including additional genomic drivers such as gene conversion. For *C. remanei*, some of these challenges might be overcome by including broader sampling from across the entire species range so that local demographic differences do not unduly influence the core species-wide diversity patterns generated by conserved genomic processes such as patterns of recombination and mutation. It is equally likely that the sensitivity of this approach can be enhanced when we are able to add in additional complexities such as realistic demographic histories and local spatial and temporal dynamics to the simulations.

In this additional analysis, we were able to classify evolutionary forces that shaped diversity in simulated populations, applying this approach to empirical data will require more comprehensive simulations of realistic biological scenarios and additional high-quality empirical genomic data.

References

1. Min S, Lee B, Yoon S. Deep learning in bioinformatics. Brief Bioinform. 2016; bbw068. doi:10.1093/bib/bbw068
2. Zhang Z, Zhao Y, Liao X, Shi W, Li K, Zou Q, et al. Deep learning in omics: a survey and guideline. Briefings in Functional Genomics. 2019;18: 41–57. doi:10.1093/bfgp/ely030
3. Krassowski M, Das V, Sahu SK, Misra BB. State of the Field in Multi-Omics Research: From Computational Needs to Data Mining and Sharing. Front Genet. 2020;11: 610798. doi:10.3389/fgene.2020.610798
4. Ching T, Himmelstein DS, Beaulieu-Jones BK, Kalinin AA, Do BT, Way GP, et al. Opportunities and obstacles for deep learning in biology and medicine. Journal of The Royal Society Interface. 2018;15: 20170387. doi:10.1098/rsif.2017.0387
5. Piccialli F, Somma VD, Giampaolo F, Cuomo S, Fortino G. A survey on deep learning in medicine: Why, how and when? Information Fusion. 2021;66: 111–137. doi:10.1016/j.inffus.2020.09.006
6. Sheehan S, Song YS. Deep Learning for Population Genetic Inference. PLOS Computational Biology. 2016;12: e1004845. doi:10.1371/journal.pcbi.1004845
7. Schrider DR, Kern AD. Supervised Machine Learning for Population Genetics: A New Paradigm. Trends in Genetics. 2018;34: 301–312. doi:10.1016/j.tig.2017.12.005
8. Martín Abadi, Ashish Agarwal, Paul Barham, Eugene Brevdo, Zhifeng Chen, Craig Citro, et al. TensorFlow: Large-Scale Machine Learning on Heterogeneous Systems. 2015. Available: https://www.tensorflow.org/
9. Seidel HS, Rockman MV, Kruglyak L. Widespread Genetic Incompatibility in *C. elegans* Maintained by Balancing Selection. Science. 2008;319: 589–594. doi:10.1126/science.1151107
