# Supplementary material for: Genomic diversity landscapes in outcrossing and selfing Caenorhabditis nematodes
Source: PLoS Genet. 2023 Aug 16;19(8):e1010879. doi: 10.1371/journal.pgen.1010879 (PMC10461856; doi:10.1371/journal.pgen.1010879)
Supplement: Attachment — Submitted filename: Response_to_reviewers_PLoS_C.remanei_popgen.pdf [file pgen.1010879.s020.pdf]

Dear Drs. Henderson and Bomblies,

Thank you for the thorough review of our manuscript and the many constructive criticisms provided by both you and the reviewers. We have sought to address each of these concerns. The revised version of the manuscript has undergone substantial improvement, including reorganization and the separation of the deep learning section as a Supplementary Appendix. As a result of these changes, we believe that the readability of the manuscript has improved considerably. Please find the reviewers' comments in bold below, along with our responses in regular face. We provide an additional version of the manuscript with all the changes highlighted in color to make it easier for the reviewers and editors to track the revisions. We hope that you will find the current version now acceptable for publication in PLOS Genetics.

## Response to Reviewer #1, Mark Blaxter

**General**

**I really liked this MS and think the data and the analyses are (in large measure) important and well performed. I have an issue with the AI modelling component which seems under-described and under-performs. The core findings - that genome-wide diversity, LD and related measures are all indicative of higher effective population sizes in CaeRema, and that the arms-and-centres structure identified in CaeEleg is present in CaeRema and is likely driven by the same recombinational landscape - are important, and show the impact of selfing on CaaEleg and especially the impact of linked selection/sweeps. These findings are not _unexpected_ as single genome assemblies of several Caenorhabditis [and other] species have already shown similar strong patterning on chromosomes, but they are significantly deepened by the population genomic analysis of CaeRema. While this is currently a 2-way comparison (and thus could be significantly biased by unknown factors impacting either species) the analyses are well performed and - given that they conform to much theoretical inference - are likely to be very strong.**

> We would like to express our deep appreciation for the comments you provided and the thoughtful work you put into reviewing our manuscript!
>
> One of the major changes in the current version of the paper is that we moved the classification section into Supplementary Data Appendix A1. We agree that this aspect of the original analysis was inconclusive and hope that such an approach can be improved in the future. The overall flow of the manuscript has significantly improved without this section.

**One of the critical findings from the work of the Andersen lab recently has been the**

**discovery and definition of a large number and span of highly divergent regions in CaeEleg where short reads do not map. This generates a false run of homozygosity against the reference if the reference region(s) are present, or an uncovered gap if alternative regions are present This structure is significant (up to 20% of the genome is affected.) Is similar structure present in the CaeRema cross parents, or in the resampling data (perhaps particularly in the divergent individuals)?**

> We agree that hyper-divergent regions are problematic for diversity metrics in for *C. elegans* and potentially *C. remanei*. We consider coverage in all our estimations in diversity statistics. In this case, the diversity estimates are at the lower bound. The percentage of affected genome in *C. remanei* is similar to that in *C. elegans* (20-25%). As most of these divergent regions located in repetitive regions, we hope that this problem will be resolved in the future, for instance, by usage of long read data. We added a comment to recognize this potential issue in paragraph 642ff:
>
> "… It remains an open question if such hyper-divergent regions are present in populations of outcrossing *Caenorhabditis* species, because short-read data, such as that used here for *C. remanei* tends to be difficult to align in hyper-divergent regions of high recombination rate. This can potentially lead to a lowered estimation of diversity, as regions with poor coverage were excluded from the analysis. Long-read data will be required to close these gaps in the genomic coverage."

**If present, defining it might assist in partitioning analysis of variation to only deal with regions of the reference that can be/are expected to be covered, and thus can have credible allele calls. The comment at line 969 makes me wonder if just this process might be at play here.**

> We completely agree that bioinformatic choices and uneven coverage can contribute to the differences between simulated and empirical data. In our study, we took the coverage into consideration while making the estimations. However, we believe that the primary differences relative to simulations likely arises from demographic complications that we couldn't include in the simulations due to computational limitations. We hope to explore this aspect in future studies.

**The modelling is interesting in showing the interacting impacts of different population level processes (neutral and adaptive) in patterning the diversity along**

**chromosomes, and is a useful extension of the inferences drawn from the population genomic data. The statement "as predicted by theory" rings loud here - what new or surprising is present in these analyses and what does the contrast between CaeRema and CaeEleg bring to the discussion. The text here could be shortened significantly in this context.**

One of the main objectives in this study was to characterize the chromosomal context of empirical patterns of diversity in outcrossing nematodes to contrast with selfing species. To our knowledge, it is the first study that demonstrated whole genomic landscape of diversity in animals that are phylogenetically close, have similar genomic organizations, and have very different reproductive modes. The references to theoretical concepts were essential in understanding and interpretation of our results, especially the expectations focused around single nucleotide differences, which have been the primary target of most theoretical work, and the interaction of genetic processes and mating system in the light of whole genome structure, which is the focus here. We have shortened the text to better highlight observations that are more novel in this regard.

**The convolutional neural network component of the work is the weakest and I would suggest that it is removed from the MS. As I understand it the authors used a set of parameters derived from the empirical data to generate a set of synthetic data on a set of model chromosomes, and then back tested the emergent classifier on the real data. The classifier performed poorly, suggesting that the real CaeRema data derived from a selfing population rather than a sexual one. While it is important to publish negative results, I am not convinced that these constitute real negative data - they suggest that the CNN was not able to extract enough information from the model chromosomes which in turn suggests the modelling was not modelling real world processes (as we assume that the measurement of CaeRema biology - ie outcrossing predominates in a some what closed and thus inbred population - is correct).**

We agree that the classification performed poorly on empirical data in the situation that we explored. We removed the classification sections from the introduction, results, discussion, and methods, and placed them in Supplementary Data Appendix A1. We believe that this change has improved the readability of the paper, made it easier to follow the logical flow, and better emphasizes our novel results. Although the classification results are inconclusive, we still think it is best to keep them as a supplementary part of the manuscript. Otherwise, its absence raises questions about the possibility of using our simulations to interpret empirical observations. Through this additional analysis, we showed that to achieve this goal, improvements in both the quality of the data and the realism of the simulations are necessary, and we hope to address these issues in future research (particularly with the forthcoming pre-release of a more powerful parallel version of the SLiM framework that we used in the evolutionary simulations).

**Analytic comments**

**The CaeRema and CaeEleg genomes are different sizes (~25%) and it is thus of interest to ask where this difference lies. This analysis impacts on analyses that compare the "proportion" of each chromosome that is in each partition (arm; centre; arm). My calculations indicate that the centre partitions in both species have very similar spans, and that the expansion in CaeRema [or shrinkage in CaeEleg) is largely due to change in the arm partition spans. In the context of discussions of what drives the differences in patterns of evolution between the species this is I think germane.**

We agree with this assessment and added a corresponding comment on line 182.

**Similarly, it would be good to know the arms vs centres distribution of the ~56% of the CarRema genome for which ancestral states could be inferred: my suspicion is that these 56% are biased to the centres, and this might impact analyses (though from Fig 3 the contrasts are so clear that I suspect that any impact might be minimal).**

We have estimated the fraction of the inferred ancestral sites within domains and about half of them located on the arms (line 274).

**In lines 278ff it is mentioned that a "clearer definition" of the boundaries of arms and centres was possible. Does this mean that the numerical base position of the boundaries was changed because of this "clarification"? If not, it is not clear what clarification was afforded.**

We agree that this sentence was a little confusing. We have rephrased it (line 285):

"…The longer divergence time between *C. latens* and *C. remanei* allowed for resolution of population genetic processes (fixation) that are ongoing within the Toronto population (segregating variation), which resulted in more pronounced differences between the domains of the divergence landscape compared to the diversity landscape, especially near domain boundaries (Fig 3A, 3C vs Fig 2)."

**lines 308ff It is not clear what analysis is being reported. Is this the reference genome vs the imputed LCA?**

That was the reference genome (PX506) versus the imputed ancestral states. We have rephrased the sentence for clarity (lines 318ff):

“Using the inferred ancestral states and the *C. remanei* reference genome (strain PX506), we estimated the rate of nucleotide substitution and found that all types of substitutions show a consistent pattern across the chromosomes (S3B Fig). …”

**Do the authors have an explanation for the strong difference in tr/tv ratios? Why would the between species differences be so different from the within species ones for this metric? I would have liked to see a discussion of the impact of using a population of CaeRema and a single reference CaeLate in imputing the ancestor: is this the source of the difference?**

We performed additional estimation to check the consistency of our results across the genome using 1 Mb windows. The transition/transversion ratio estimations were consistent along the genome for both cases (within the Toronto population and between *C. remanei* and *C. latens*' genomes), and they were significantly different from each other. We suspect that this could be attributed to the difference in mutation spectra in *C. latens* used in the imputation. We have rephrased and expanded this section (lines 320ff):

“… Transition over transversion bias with standard deviation estimated in 1 Mb windows in this comparison was 1.16±0.1, which was smaller than the 1.5±0.1 ratio observed within the *C. remanei* population sample from Toronto (Kruskal-Wallis $\chi^2 = 306.94$, *P*-value $< 10^{-16}$). The difference in biases may be attributed to shifts in the mutation spectrum ([102]) within the *C. remanei* population, as well as disparities in the mutation spectrum of *C. latens* used in the ancestral inference. …”

**Minor questions**

**Tab3 line 398**

**What does "low accuracy" mean in the methods column? Is it a statement on the performance of the method class as a whole (in which case remove) or a particular form of coalescent analysis (in which case we need a definition).**

We have removed the note from the table. It is a general statement that coalescent methods tend to be less accurate for more recent generations.

**Textual comments**

**There are several textual infelicities in the MS which make reading somewhat difficult in places. Some are (imho) grammatically wrong, others I think obscure the meaning and content.**

**I found the opening paragraphs of the introduction over-general, and sometimes hard to parse scientifically. This could be shortened and focussed. The general statement that there are both selective and neutral processes at work, and that these processes overlap with each other in their impacts on the genome, and that general structural features of the genome - driven by selection on structure in general - can have consequences for/impose constraints on realise change, is unproblematic... but is more textbook introduction than research paper introduction.**
**eg line 48 "factors act and constantly interact"? Not sure of the meaning of this phrase.**

We have condensed that paragraph (lines 59ff).

**The authors close the legends of (most) figures with a narrative discussion of what the figure is being used to show. My style is to leave the figures to show data and to have such narrative in the main text. I am not sure what PLoS protocol is.**

We appreciate your suggestion. We have removed most of them, but have left a few for which this language helps in the interpretation of the figure itself.

**Other text / presentation notes**

*** arms and centres are defined twice, and the definitions suggests capital-A Arms and capital C-Centres are the short forms (which are not used again).**

Thank you! We removed the second definition.

*** I would suggest moving the lists of values/means, p-values, SDs etc from section "Genetic diversity.." [lines 186ff: lines 194ff, 208ff, 222ff, 227ff] to a table where the hypothesis tested, the statistical test and the list of values could be presented accessibly. The current extended sentence listing all values is hard to read and obscures the statement of the actual findings.**

We have moved the listed statistical comparisons to Table 2. We agree that the original sentences were difficult to follow and appreciate your suggestion.

**I would also suggest using the same exponent for all measures within a set/comparison (eg if values are 1.2 x10-2 and sd is given as 2.3 x 10-3 thuis suggests greater accuracy/significant figures for the SD. Give sam number sig figures for each, and think about normalising between species where one is x10-2 and the other x10-3)**

We normalized the values (lines 205, 218, 382, Table 3 and Table 4).

*** the plots of metrics across the genomes are stated to be measured in sliding windows of 100 kb (mostly) [eg Fig 2, Fig 3]. Is this really a sliding window? in which case what were the step sizes? or is it a tiled window of 100 kb (which is equivalent to a sliding window of 100k with a step of 100k, but, I would contend is categorically different - no sites are counted > once in a step window, whereas they are in a sliding window)**

Thank you for bringing this to our attention. We used non-overlapping windows in most cases, and we have updated the language throughout the manuscript (Fig 2,3,4,6, and in the Methods section).

*** I have a campaign to halt the use of the word "worm" when nematode is correct (and there are so many other worm-like taxa). Perhaps the authors could look at the ~9 instance of the use of worm(s) and decide if nematode(s) would be better.**

We agree and have made changes to use 'nematodes' whenever possible.

*** spans are sometimes presented as "100-kb", and sometimes as "100 kb". I think 100 kb is correct in each case. There are a few instances of no separation between value and unit**

We have changed all instances to the version with a space.

*** sometimes "ten times" is spelt out, sometimes it is given "10x" [etc]; use the spelt out version**

We opted to use the spelled-out version whenever it was feasible.

*** line 232 "recombination rate IN"**

Thank you for pointing out, we have changed it (line 381).

*** line 239 location of "details" missing**

We added the missing reference (line 244).

*** occasional flipping between past tense and present tense in reporting of work done / results found (even in same sentence) - see lines 354ff for eg.**

After careful review, we have tried to ensure that we are consistent in our use of tense. We focus on things that we *did*, the outcomes that we *find*, and the state of nature that *is*.

*** line 340 is this a new paragraph, or just a misplaced carriage return?**

We combined it to one paragraph (lines 356ff).

*** line 440 data "show" (data are plural)**

Thank you for pointing out this error. We have corrected it on line 460.

## Response to Reviewer #2

**Taking advantage of the Caenorhabditis clade, where selfing has evolved independently three times, Teterina et al use genomic analyses and evolution simulations to provide insight into the contribution that selfing vs. outcrossing has on genetic diversity. The authors find that while the overall pattern of diversity mirrors the underlying pattern of genetic recombination on chromosome arms vs. centers in both C. elegans (selfing) and C. remanei (outcrossing), C. remanei has significantly higher levels of genetic diversity as is predicted for an outcrossing species. This is an important study that sets the stage for future work to elucidate contributions of reproduction, selection and demography to genetic diversity. The results will be of interest to the broad audience of PlosGenetics.**

We thank you for recognizing the value of our study.

**1. The authors should consider making the manuscript more assessable to those outside population biology – they often mention programs, parameters, and values that have little meaning to those outside the field.**

Thank you for your suggestion. We have removed a significant portion of the classification and related discussion from each section of the manuscript and included it in the Supplementary Data Appendix A1. We believe that this change will improve the flow and readability of the manuscript.

**2. The authors should provide more information in the introduction about what is known about selfing vs. outcrossed species. There are some very interesting genetic studies from the Haag, Schedl and Ellison labs that show only a couple of mutations are required to convert between these two different reproductive modes.**

> We appreciate your suggestion and find it a great addition to the point of why *Caenorhabditis* nematodes are one of the best possible animal models to study the effects of reproduction. We have added it to the introduction (lines 115ff):
>
> "… *C. elegans* is a model species for behavior genetics ([53], [54], [55], [56]), development biology ([57], [58], [59]), and experimental evolution ([60], [45], [61], [62]). Moreover, the occurrence of hermaphroditism in *Caenorhabditis* nematodes can be attributed to changes in a few pathways ([63], [64], [65], [66]), and the sex determination can be genetically manipulated, making *Caenorhabditis* species an unprecedented tool for study of the effects of reproduction mode in metazoans ([67], [68] [69], [45], [70]). …"

**3. I understand that this is outside the scope of this study, but it seems a more powerful approach would be look at more than one self vs. obligate outcrossing species to see whether the differences observed here are more generalizable. In the absence of this, the authors should be a little more careful in not generalizing their results beyond the two strains they examined.**

> We appreciate your input and acknowledge that various factors can influence diversity landscapes, and that comparisons of multiple species and/or populations would be more informative. This was partially the reason we used simulated populations, as they allowed us to obtain expected distributions of diversity statistics. We very much look forward to future studies that will provide data of this nature for additional species, possibly encompassing other nematodes or animal systems with different modes of reproduction.

## Response to Reviewer #3

**This paper provides some important findings for C. remanei, including a genetic map and a polymorphism/LD survey of natural isolates. The species is of broad interest because it is an outcrossing relative of C. elegans, and because of the strikingly high level of nucleotide diversity found in natural populations. The authors also pair the new data with a reanalysis of existing data for a C. elegans population, and the contrast between the two is informative. Overall the genetic**

**maps between the two species are qualitatively similar, and the drastic polymorphism and LD differences between the species (which were previously discovered), are consistent with the major life style difference (i.e. remanei is an outcrossing species whereas elegans reproduces primarily by self-fertilization).**

We thank the reviewer for their overall positive evaluation of our study and thoughtful comments.

**One significant puzzle is not explained in the paper, and really needs to be addressed more fully. The inbreeding coefficient for C. remanei was found to be 0.38, a strikingly high figure for an outcrossing species. The PC plot (fig S4) does not really help us understand the cause of this, and neither does the corresponding text " This cluster was most likely formed from individuals from a single-family lineage displaying intensive". There are a couple things about the Fis value that need attention here. (1) Are there runs of autozygosity consistent with consanguineous mating? (2) How long ago did the inbreeding occur? (3) How could inbreeding have occurred if the individuals mostly came from different isopods? (4) Were the sampled isopods from different species?**

We agree that the high inbreeding coefficient for *C. remanei* is an intriguing finding that deserves more attention. (1) We did not observe long runs of homozygosity in that sample. (2) From our understanding, the observed level of diversity and such $F_{is}$ values would be consistent with a very few, 2-5, generations of inbreeding of siblings or half-sibs (Falconer 1989, Table 5.1). We have added a corresponding comment (lines 355ff) to clarify this:

"… This view was supported by relatively high $F_{is}$ values along the *C. remanei* genome (0.38 ± 0.15, S2M Fig). Such a value can be obtained through just a few generations of sibling mating (see Table 5.1 in [110]), which brings new insights into the biology of *C. remanei*. …"

(3) All except two of the individuals were isolated from different isopods (lines 971ff):

"… One pair of *C. remanei* individuals derived from a shared isopod host (NS50-1, NS50-2), whereas all other individuals were isolated from different isopods."

(4) All isopods were the same species.

**Lastly, regarding the high Fis, could there be a sequencing or pipeline problem that**

**missed a lot of heterozygotes? The methods are thin regarding this possibility, and appear to not even tell us what the read depth was (sorry if I missed it somehow).**

We were also surprised by the high inbreeding coefficient values observed for outcrossing *C. remanei*. However, we found that these values were consistent across the genome (S2M Fig) and were not affected by coverage. Moreover, $F_{is}$ values estimated from simulations were as expected (S10N Fig), suggesting that the $F_{is}$ values in *C. remanei* were not an artifact of the estimation method. It is worth noting that the *C. remanei* sample was collected from a single location, so it is possible that this finding reflects some features of the nematode biology and population structure that are, as yet incompletely understood, and it seems that the occasional inbreeding can occur.

We have added a comment on the depth of coverage to the Methods (line 987):
"… The individuals included in this study had an average depth of coverage ranging from 20x to 40x. …"

**The analysis of changing population size on pp 16-18 and figure 5, is confusing. The two parts of figure 5 seem to tell different stories over recent times, and in particular the SMC++ suggests a flat history for everyone back to 1000 generations ago (albeit with a wide variance depending on the replicate). This is not consistent with the LD based story, nor the text. On balance it seems these analyses just are not working well, and it might be best to just report the conflicting signals, along with a figure of the SFS.**

We agree that the history inferred by the two methods not fully consistent, in part due to different sensitivities of the methods to different time frames. In particular, the coalescent method tends to be less accurate when examining very recent or very ancient (> 4Ne) time periods. To better acknowledge this issue, we added shading to Fig 5 to represent the regions of greatest uncertainty. We added additional comments about the shading to the Fig 5 description:

"… The grey shading on the left-hand side of the plot indicates the regions of recent demographic history where estimations using this method may be less reliable and inconsistent with LD-based estimations, which are more appropriate for recent historical events. The grey shaded areas on the right-hand side of the plot show

the regions where the inference should also be less accurate as we move further back in time beyond the threshold of ~4Ne generations. …”

**Unfortunately, the simulations and the neural network analyses add very little to the paper. The problems here start with the motivating rationale, which was to simulate data under a wide array of population genetic models to look for the kinds of model differences that mirror the observed differences seen between the elegans and remanei data. This kind of approach, without specific questions, and without much use of data driven estimators, seems bound to provide results that are hard to interpret.**

Following the reviewers’ advice, we moved the bulk of the simulation analysis to the Supplementary Appendix A1 (see also response to reviewer #1). We think that this change improves the clarity and flow of the manuscript.
