# Supplementary material for: Genomic diversity landscapes in outcrossing and selfing Caenorhabditis nematodes
Source: PLoS Genet. 2023 Aug 16;19(8):e1010879. doi: 10.1371/journal.pgen.1010879 (PMC10461856; doi:10.1371/journal.pgen.1010879)

Dear Drs. Henderson and Bomblies,

We are thankful for the evaluation of our manuscript. In the updated version of the manuscript, we have addressed of all of the reviewers' concerns. We hope that the current version of the manuscript will be acceptable for publication in PLOS Genetics.

## Response to Reviewer #2

**The revised manuscript by Teterina et al presents genetic and genomic analyses and evolution simulations to provide insight into the contribution that selfing vs. outcrossing has on genetic diversity. The authors find that while the overall pattern of diversity mirrors the underlying pattern of genetic recombination on chromosome arms vs. centers in both C. elegans (selfing) and C. remanei (outcrossing), C. remanei has significantly higher levels of genetic diversity as is predicted for an outcrossing species. Evolutionary simulations provide insight into contributions of selection, recombination, mutation, and selfing on genetic variation. The authors have done a good job addressing the previous reviews and the results will be of interest to the broad audience of PlosGenetics.**

We appreciate the feedback and grateful for the provided insights.

## Response to Reviewer #3

**This paper is improved.**

We appreciate your feedback and we attempted to address any potential concerns.

**However I remain concerned about the estiamtes of historic population size, and apparent inbreeding.**
**There seems to be a good chance that something fairly fundamental about the history of the sampled population has been overlooked. There are two main clues to this. First are the analyses of historic population size. As mentioned previously, the two kinds of analyeses shown in the figure have little in common. Both can't be right. Coloring figure 5 does little to alleviate the discrepancy.**

We agree that the inferred demographies obtained by these two algorithms were inconsistent and have been pursuing potential causes. We consulted with colleagues who have tested various approaches to infer the demographic history

of the populations using simulations that included demography, and based on their recommendations decided to remove the Fig5 A analysis (GONE). In general, we have lost confidence in the ability of this method to inform population history accurately. However, we retain the region of shading in the SMC++ plot as it indicates the potential for lower accuracy at certain time scales. Fig 5A and all results associated with this method have been removed from the text. This elevates the discrepancy indicated by the reviewer and are grateful for the push to dig deeper into this, as it helps head off a potential issue and clarifies the paper as a whole.

**Second, there is the high level of Fis. The author's suggest recent consainguineous mating, which could certainly do it. However this would also leave a high variance in Fis along the chromosomes, something which was not observed. Again, something is amiss. The paper is informative on the mapping and polymorphism fronts, but the population genetic analyses are incomplete.**

We agree that recent inbreeding should leave a variance in $F_{is}$ along the genome, which aligns with our observations. We want to clarify that our previous response referred to consistently high $F_{is}$ values, indicating the average high value across the genome. We include below a panel from our supplementary figure (S2M Fig), which demonstrates substantial variance in $F_{is}$ values along the chromosomes as suggested by the reviewer. Please note that this was already included in the previous submission. Also, we estimated $F_{is}$ using various bioinformatic parameters, however, all resulted in high average $F_{is}$. Given that the Toronto sample was collected from a single location, it appears plausible that nematodes in this population may have undergone recent inbreeding. Hopefully, we will have more samples in the future to enhance our understanding of *C. remanei* biology. We hope that this clarification will help to resolve the concern.

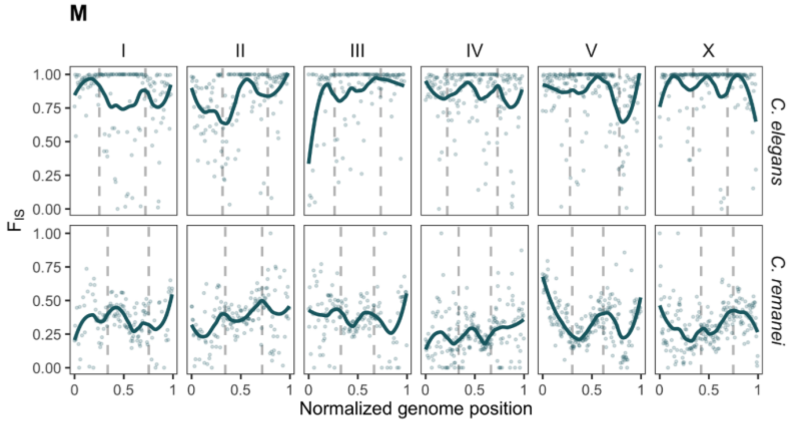

Supplement: Attachment — Submitted filename: Response_to_reviewers_second_revision.pdf [file pgen.1010879.s021.pdf]
